# Granulomatosis with polyangiitis: a 17 year experience from a tertiary care hospital in Pakistan

**DOI:** 10.1186/s13104-018-3434-2

**Published:** 2018-05-16

**Authors:** Omar Irfan, Haris Khan, Zarrar Khan, Alina Ashraf, Rimsha Ahmed, Javaid Ahmad Khan, Ali Bin Sarwar Zubairi

**Affiliations:** 1 Department of Medicine, Aga Khan University, Stadium Road, Karachi, 74800 Pakistan; 2 Department of Basic Sciences, Aga Khan University, Stadium Road, Karachi, 74800 Pakistan; 3 Medical Student, Aga Khan University, Stadium Road, Karachi, 74800 Pakistan; 4 Department of Medicine, Aga Khan University Hospital, Stadium Road, Karachi, 74800 Pakistan

**Keywords:** Granulomatosis with polyangiitis, Vasculitis, c-ANCA, p-ANCA

## Abstract

**Objective:**

Granulomatosis with Polyangiitis (GPA) is an autoimmune, multi-system, small and medium vessel vasculitis with granulomatous inflammation. Aim of this study was to assess the clinical and radiological presentations of patients with GPA amongst the Pakistani population. It is a single centre retrospective single observation study.

**Results:**

Study was conducted at the Aga Khan University Hospital, Karachi with records were reviewed from January 2000 to December 2017. Definitive diagnosis was made using a combination of serological anti-neutrophil cytoplasmic antibody (ANCA) testing along with the clinical and radiological presentation. A total of 51 patients met the diagnostic criteria in the time frame of the study. There were 23 males and 28 females with mean age of 44.0 ± 17.8 years on presentation. Arthritis was the most common symptom present in 41.2% of the cases followed by cough in 32.0%. Sixteen patients showed pulmonary infiltrates on chest X-ray. C-ANCA was positive in all of the patients compared with 21.6% p-ANCA positivity. A total of 13 biopsies were done. The median Birmingham Vasculitis Activity Score was 12. We report a 17.6% mortality rate with 5 deaths occurring due to respiratory failure. GPA is a diagnostic challenge leading to late diagnosis which can contribute to significant morbidity and mortality specially in the Third World.

## Introduction

GPA is an autoimmune small vessel vasculitis associated with anti-neutrophil cytoplasmic antibodies (ANCA). The clinical manifestations include systemic necrotising vasculitis, necrotising glomerulonephritis and necrotising granulomatous inflammation. GPA was first described in the literature in a case report in the late nineteenth century and was previously known as Wegener’s Granulomatosis [1]. While it is uncommon overall, a significant geographical variation has been observed in the prevalence of this disease. A latitudinal gradient has been identified with increasing trend in prevalence as the distance from the Equator increases [2].

In GPA, ANCA is mainly directed against Proteinase 3 (PR3); there is strong evidence from various in vitro studies that ANCA plays a crucial role in the mediation of small vessel vasculitis [3]. GPA usually starts as a localized granulomatous inflammation of the respiratory tract that later generalizes into small vessel vasculitis [4, 5]. Older age, pulmonary and kidney involvement are features associated with poor prognosis and increased mortality [6]. The first case of GPA was reported by a German clinician, Heinz Klinger in 1931 who believed it to be a variant of polyarteritis nodosa, but it was Friedrich Wegener who proposed this as a separate pathology based upon the post mortem findings seen in three of his patients [7, 8] Several large case series on GPA have been published from various countries, with the largest series reporting 445 patients from Germany [9]. This is the first large institutional experience on the clinical manifestations of GPA patients amongst the Pakistani population. We report a series of 51 cases from our institution. Our study focuses on the clinical and imaging features of GPA with the hope that it will assist clinicians in better identifying and managing this condition. We also summarize the compare the clinical features of our patients with those from other ethnic group studies from literature in a tabulated form.

## Main text

### Methods

A retrospective, observational study was conducted at the Aga Khan University Hospital (AKUH), Karachi. Records of all patients diagnosed with GPA from January 2000 to December 2017 were reviewed. Age, gender, clinical features at the time of diagnosis, laboratory and histo-pathological findings, imaging, type of treatment, response to treatment and complications were collected in a predesigned questionnaire. The files were retrieved using international classification of diseases-9 (ICD-9) coding. No experimentation on Human and animals was done. This study was approved by the local ethical committee of the institution. SPSS IBM version 20 was utilized to analyze the data. Each categorical item from the questionnaire was summarized by frequency count and percentage.

We used non-standardized criteria to diagnose the disease. The 1990 American College of Rheumatology and 2012 Chapel Hill Consensus Conference criteria for the classification of the vasculitis have been accepted [6], however they are not intended to be used as diagnostic criteria. The ACR criteriawas devised before the widespread recognition of microscopic polyangiitis as a separate disease as well as prior to recognition of antineutrophil cytoplasmic autoantibody (ANCA) testing as a significant diagnostic tool. Therefore being a non invasive test, we used serological evaluation as the initial step for diagnosis of GPA at our institution. C-ANCA is known to have sensitivity of 91% and specificity of 99% for GPA [7]. Birmingham Vasculitis Activity Score version 3 (BVAS-v3) was used to determine the disease activity. Outcomes recorded were death, cause of death and number of relapses.

### Results

During the study period, 51 patients diagnosed with GPA were identified. The mean age of presentation was 44.0 ± 17.8 years with 23 males and 28 females. The respiratory system was most commonly affected in 41 (80.4%) patients, followed by renal in 28 (54.9%), Ear Nose and Throat (ENT) in 25 (49.0%), musculoskeletal system in 22 (43.1%), nervous system in 9 (17.6%), integumentary system in 8 (15.7%) and ocular system in 6 (11.8%) patients. Arthralgia was the most common presenting symptom seen in 21 patients followed by cough in 16 patients. The clinical symptoms on presentation are as shown in Table 1.

**Table 1:** Clinical manifestations of the patients with GPA

Clinical manifestations	Frequency (n)	Percentage (%)
ENT	25	49.0
Rhinosinusitis	6	11.8
Mucosal ulcers	1	2.0
Epistaxis	7	13.7
Hearing loss	9	17.6
Collapsed nasal bridge/saddle nose deformity	2	3.9
Pulmonary	41	80.4
Cough	16	31.9
Hemoptysis	9	17.6
Chest discomfort	7	13.7
Dyspnea	5	9.8
All	4	7.8
Renal	28	55.0
Hematuria	11	21.6
Renal failure	17	33.3
Ocular	6	11.8
Conjunctivitis	4	7.8
Episcleritis/uveitis	2	3.9
Neurological	9	17.6
Cranial nerve palsies	4	7.8
Peripheral neuropathy	5	9.8
Musculoskeletal	22	43.1
Arthritis/arthralgia	21	41.2
Myalgia	1	2.0
Cutaneous	8	15.7
Purpura/ecchymosis	6	11.8
Ulceration	2	3.9

All of the selected patients underwent both p-ANCA and c-ANCA on initial work up. The c-ANCA was positive in all patients whilst only 11 patients were positive with p-ANCA. Thirty three patients had a raised ESR (> 20 mm/h). The radiological findings are outlined in Table 2.

**Table 2:** Radiological manifestations of the patients with GPA

Parameters	Frequency (n)	Percentage (%)
Chest X-ray findings	51	100
Pulmonary infiltrates	16	31.4
Diffuse alveolar hemorrhage	9	17.6
Nodules	9	17.6
Cavitations	9	17.6
Pleural effusion	4	7.8
Normal	4	7.8
Chest CT findings	18	35.3
Nodules or masses	2	3.9
Airspace consolidation	6	11.8
Cavitation	5	9.8
Ground-glass Appearance	1	2.0
Bronchiectasis	4	7.8

The most common finding on chest X-ray was pulmonary infiltrates seen in 16 patients (31.4%), followed by pulmonary nodules, alveolar hemorrhages and cavitations in 9 patients each. A total of 13 biopsies were done which included 7 renal, 3 nasal, 2 lung and 1 skin specimen. Four renal biopsies showed crescenteric glomerulonephritis, while focal necrotizing glomerulonephritis was noted in 3 specimens. Chronic granulomatous remodeling along with necrotic change was well appreciated in all the nasal biopsies. A mixed picture of acute and chronic inflammation with scattered multinucleated giant cells and vasculitis could be identified in the lung and skin biopsy results.

Forty five patients received a pulse dose (1 g/day) of intravenous methyl prednisone. Remission was achieved in 32 patients while 9 patients relapsed. Prednisolone was the most common agent used for induction therapy in 32 (62.6%) patients followed by cyclophosphamide in 11 (21.6%) patients. Other therapies include Methotrexate, Rituximab, Plasma exchange and Azathioprine. Prednisolone was the most common agent used for maintenance therapy in 41 (80.3%) patients followed by azathioprine in 8 patients (15.7%). The complications that developed after the initiation of treatment were divided into 3 categories: opportunistic infections, malignancy and cytopenia. Sixteen patients developed opportunistic infections. Malignancies and cytopenia were noted in four each. Nine patients passed away during the study period; of these 5 deaths occurred due to respiratory failure, 3 deaths happened due to sepsis and 1 patient succumbed to heart failure. The median BVAS-v3 score at presentation among all patients was 12.0 with range from 4.0 to 24.0. BVAS-v3 score was higher among patients who expired compared to those who survived (14.1 vs. 11.1, P > 0.05). However the difference was insignificant therefore we cannot conclude if the BVAS-v3 score at presentation is useful in determining severity of disease and in determining prognosis.

### Discussion

This study, documenting 17 years of experience, is the most comprehensive, single-institution study describing the clinical and radiological characteristics, management, and outcome of patients with GPA from the Pakistani population. Respiratory manifestations were recorded in 80% of our patients as compared to 50% involvement observed in the literature whilst alveolar hemorrhage, lung nodules, and respiratory failure were the key presenting features of this condition [8]. The second organ system most commonly involved includes the kidneys with focal segmental necrotizing glomerulonephritis being the most consistently presenting feature [10]. More than half of the patients (55%) in our study had renal manifestations and one-third of them developed renal failure. Our study reports 49% of the cases presenting with ENT manifestations. Hearing loss and epistaxis were the most frequent symptoms. Previously, more than 70% of cases have been reported to have presented with signs related to ENT ranging from crusting rhinorrhea to damage of the facial cartilage causing deformities [11]. Other rare symptoms involved the mucocutaneous, nervous and ocular systems.

On radiological evaluation, 16 of 51 patients demonstrated pulmonary infiltrates in the form of consolidations making it the most common presentation in our setting. Cordier et al reported pulmonary infiltrates to be the second most common presentation making up 53% of the patient population in their study [12]. In 1990 the American College of Rheumatology endorsed a criterion for the diagnosis of GPA. One of the clauses of the criteria was the presence of abnormal radiological findings on chest X ray [13]. Chest CT is considered superior to chest X-ray in the diagnosis of small vessel vasculitis [14]. Out of the 51 patients, 18 had an HRCT done. Six out of 18 patients showed airspace consolidation, making it the most common finding. Multiple studies have shown that abnormal radiological findings are found in 50–75% of the population with GPA [15] with nodules and masses being the two most common radiographic manifestations. Studies have also labeled the presence of bronchiectasis as a rare presentation [16] as supported by the presence of bronchiectasis in only 4 patients in our study.

Moving on to serological investigations, ANCA testing has been employed as diagnostic criteria for GPA at our institution. Anti-neutrophilic cytoplasmic antibody (ANCA) sensitivity can be as low as 60% in localized disease [17] whereas ANCA is both sensitive and specific for GPA being present in 80–90% of patients with systemic disease. Still, there are some reported cases where the ANCA was negative [18]. A previous study by Chloe et al showed an 80% c-ANCA positivity [19]. Prednisolone and Cyclophosphamide were the most commonly used drugs for induction in 32 and 11 patients respectively. For severe disease, plasma exchange in addition to oral corticosteroids and cyclophosphamide administration has been recommended to halt the progression to end stage renal failure [20]. Oral corticosteroid therapy in conjunction with daily oral cyclophosphamide or intravenous pulsatile cyclophosphamide is recommended for systemic disease with organ dysfunction [20]. We had 33 cases maintained with prednisolone.

A literature review revealed a relative paucity of studies available on ANCA associated vasculitis from the developing world. Our institution reported a 13 patient registry in 2004 [21]. A 20 case series of Rhinological manifestations of GPA was also reported from Rawalpindi in 2011 [22]. To our knowledge, this is the biggest experience from Pakistan. Our neighboring Country, India has reported three institutional experiences of 60, 45 and 105 patients. They also assessed the disease activity and damage through the Birmingham Vasculitis Activity Score v.3 (BVAS v. 3) [23, 24]. The Indian series have been presented in Table 3 along with other ethnic groups Table 3. Compares the summaries of GPA series from literature. A comparable prevalence of organ system involvements are seen. In our opinion, tertiary care centers need to maintain a registry of all the ANCA associated vasculitis cases so that their clinical presentation and response to treatment can be well documented for our population.

**Table 3:** Comparison of clinical features of our GPA patients with other experiences

	Mahr et al. 2013, Europe	Holle et al.[9], Germany	Reinold-Keller et al. [6], Germany	Stone et al. (2002), USA	Shobha et al. (2018), India	Kumar et al. (2015), India	Sharma et al. [24], India	Our study, 2018
Total no. of patients	396	445	155	180	60	45	105	51
Male/female %	54/46	50/50	49/51	60/40	35/25	42/58	43/57	55/45
Mean age at diagnosis	55	51	48	47	44	46	40	44
Study period	1995–2003	1996–2002	1966–1993	2000–2003	2002–2010	2009–2014	2005–2016	2000–2017
Mean duration of follow up, years	4.75	6	7	NA	4.7	NA	3.1	NA
c-ANCA/PR-3 positivity (%)	–/79	81/80	84	76/73	93	62/38	82/62	51/100
p-ANCA/MPO positivity (%)	–/11	4	3/–	11/12	7	11/7	9/7	11/21
Organ involvement (%)								
ENT	84	98	93	90	21	80	79	49
Joints	NA	73	61	81	27	42	44	43
Lung	67	60	55	75	63	49	68	80
Kidney	77	60	54	68	70	33	31	55
Eye	39	40	40	41	11	31	41	12
Nervous system	14	17	11	10	25	10	19	18
Skin	30	26	21	39	48	9	30	16
Heart	10	11	13	4	1	7	6	NA
GIT	7	3	3	7	NA	9	12	NA
Relapses,%	47	50	64	NA	13	53	31	18
Expired,%	14	10	14	NA	18	NA	17	18

c-ANCA, cytoplasmic antineutrophil cytoplasmic antibody; ENT, ear nose and throat; GIT, gastrointestinal tract; MPO, myeloperoxidase; p-ANCA, perinuclear antineutrophil cytoplasmic antibody; PR3, proteinase 3

### Conclusion

The results from our study indicate that GPA is not uncommon in Pakistan. We believe that larger studies on GPA will assist physicians in timely diagnosis and management of the disease.

## Limitations

A few notable shortcoming of our study include the small sample size making it difficult to develop significant associations between variables. Likewise, we report data from only one tertiary care hospital of the country and so the results we present cannot be generalized due to the possibility of referral bias.

## Abbreviations

ANCA: antinuclear cytoplasmic antibody
GPA: granulomatosis with polyangiitis
MPA: microscopic polyangiitis
cANCA: cytoplasmic ANCA
pANCA: perinuclear ANCA
PR3: proteinase 3
MPO: myeloperoxidase
ENT: ear nose and throat
